# Plant-Parasitic Nematodes and Food Security in Sub-Saharan Africa

**DOI:** 10.1146/annurev-phyto-080417-045833

**Published:** 2018-06-29

**Authors:** Danny L. Coyne, Laura Cortada, Johnathan J. Dalzell, Abiodun O. Claudius-Cole, Solveig Haukeland, Nessie Luambano, Herbert Talwana

**Affiliations:** 1 International Institute of Tropical Agriculture, Kasarani, Nairobi, Kenya; email: d.coyne@cgiar.org; 2 Queen’s University Belfast, School of Biological Sciences, Medical Biology Centre, Belfast, BT9 7BL, United Kingdom; 3 Department of Crop Protection and Environmental Biology, University of Ibadan, Ibadan, Nigeria; 4 International Centre for Insect Physiology and Ecology, Kasarani, Nairobi, Kenya; 5 Sugarcane Research Institute, Kibaha, Coast, Tanzania; 6 Department of Agricultural Production, School of Agricultural Sciences, Makerere University, Kampala, Uganda

**Keywords:** climate change, cropping intensification, lesion nematodes, potato cyst nematodes, root-knot nematodes, tropical

## Abstract

Sub-Saharan Africa (SSA) is a region beset with challenges, not least its ability to feed itself. Low agricultural productivity, exploding populations, and escalating urbanization have led to declining per capita food availability. In order to reverse this trend, crop production systems must intensify, which brings with it an elevated threat from pests and diseases, including plant-parasitic nematodes. A holistic systems approach to pest management recognizes disciplinary integration. However, a critical under-representation of nematology expertise is a pivotal shortcoming, especially given the magnitude of the threat nematodes pose under more intensified systems. With more volatile climates, efficient use of water by healthy root systems is especially crucial. Within SSA, smallholder farming systems dominate the agricultural landscape, where a limited understanding of nematode problems prevails. This review provides a synopsis of current nematode challenges facing SSA and presents the opportunities to overcome current shortcomings, including a means to increase nematology capacity.

## Introduction

Sub-Saharan Africa (SSA) is a region beset with numerous challenges, including the need to drastically improve agricultural productivity to support its rapidly rising population. To achieve this, numerous shifts in attitude and practice must occur, including pest and disease management. Plant-parasitic nematodes (hereafter, nematodes), in particular, are neglected relative to other pests and pathogens. A critical under-representation of tropical nematologists, compared with other disciplines, and a universally poor recognition of nematode problems underpin this shortcoming. This review focuses on the impact of nematodes across SSA and their adverse effect on crop production and sustainable intensification of agriculture. We also reflect on the regional neglect of nematology and consider broader implications for future food security. We illustrate this review with crop case studies, demonstrating the diversity and scale of nematode problems. We have taken a pragmatic approach, leaning on the personal experiences and exposure of the authors in SSA agriculture and capacity building in the region. It is not our intention to provide extensive detail of nematode pests crop-by-crop or to dwell on management options, which have been addressed elsewhere (22, 38, 46, 56, 106, 125, 132, 142), but rather to highlight the issues and complexity of the situation and to demonstrate the potential for progress.

## Background

With current food production figures, escalating urbanization, changing diets, and the impacts of a changing climate, there is much to be alarmed about in SSA. By 2050, Africa will account for more than half the growth in global population. Beyond 2050, Africa will contribute more to global population growth than any other region (53, 139). There is no doubting the need to increase agricultural efficiency and productivity. To achieve this, cropping systems must become more intensified while improving water-use efficiency, managing elevated pest and disease threats, and boosting the quality and use of external inputs (81). Indeed, every opportunity to make better use of existing water or nutrient resources should be taken, as this adds resilience in the face of climatic, biotic, and abiotic stresses while still delivering on productivity goals (82). When concluding the International *Meloidogyne* Project, Sasser & Carter (122) emphasized the need to address the obstacle of nematodes if world hunger is to be alleviated. Although there has been considerable progress since 1985, there remains an integral requirement to tackle the threat of nematode pests. The advances made in our knowledge of and ability to address nematode problems is yet to be fully integrated within SSA agriculture academic curricula or to trigger policy changes (28, 46, 132). Change is most certainly underway though, with a bright future predicted for tropical nematology (124), in part through a fundamental shift in the availability of tropical nematologists.

For the purpose of this article, SSA refers to the area south of the Sahara and, essentially, north of South Africa (**https://unstats.un.org/unsd/methodology/m49/**). Here, subsistence smallholder farming systems dominate the agricultural landscape, although larger-scale commercial farms also exist. However, there is occasion to refer to South Africa, where agriculture has been more commercialized, reliable data on nematode damage are more available, and nematode diversity and the damage they cause have parallels with SSA. Smallholder farmers may also be involved in more commercial enterprises in SSA, through their participation as out-growers to local cash crop–based industries, such as sugarcane, or to export commodity production systems, such as coffee. Although SSA is located within the tropics, it also transcends a wide range of agro-ecologies that reflect subtropical through temperate conditions because of cooler, high-altitude areas. Under these primarily tropical conditions, nematode diversity can be great and is often present as mixed-species communities, each with multiple generations per season within multiple cropping seasons per year, creating a formidable challenge to crop production (125). The impressive diversity of nematode genera and species associated with crops growing across SSA occurs as a complex community of species, making it difficult to assess the relative importance or pathogenicity of individual species. Root-knot nematodes (RKNs) (*Meloidogyne* spp.) and lesion nematodes (*Pratylenchus* spp.) are the two most important groups (75, 125) and can infect, feed on, and reproduce on an astonishing range of crops and plant species. The tropical RKN *Meloidogyne incognita* is a polyphagous species that has been considered by some as the world’s most damaging crop pathogen (137). Cyst nematodes (*Heterodera* and *Globodera* spp.) are present but as yet do not appear to be as diverse or widely distributed as RKNs, although a whole range of plant-parasitic species from other genera are also present and pose a threat to crop production in SSA.

Crop losses due to nematodes are difficult to calculate accurately, with global estimates varying considerably from $US80 billion (101) to $US157 billion per year (1). Using reliable data from the United States, nematodes were estimated to cause annual crop losses of $US10 billion, compared with $US6.6 billion for insect pest losses (62). In the United Kingdom, the potato cyst nematode (PCN) problem (*Globodera rostochiensis* and *Globodera pallida*) accounts for an estimated ˜$US70 million or 9% of UK potato production (101). There are as yet no reliable estimates of the economic losses due to nematodes in SSA.

## Nematodes and Sustainable Intensification of Agriculture

Smallholder systems are highly complex, combining a range of annual and perennial crops and cultivars in a multilayer tapestry of unsystematic randomized mixed cropping, which makes such cropping systems difficult to simplify (140). This complexity is a characteristic feature of this traditional approach to agriculture (22). Within smallholder systems, addressing nematode problems is not straightforward. Limited use of quality inputs, lack of improved techniques, insufficient access to improved cultivars, poor infrastructural networks, and poor pest and disease diagnostics prevail. Farmers and agricultural staff typically have a rudimentary understanding of nematodes and consequently lack the expertise to manage nematode infestation. Although nematodes are one of the most widespread and economically important crop pests globally (148), the evident neglect of nematology as a discipline is perplexing, especially in tropical agriculture. Nonspecific, cryptic disease symptoms and a lack of apparent damage (120) belie the importance of nematodes, which are regularly misdiagnosed or the symptoms are attributed to unknown causes, nutrient deficiency, and soil sickness (e.g., 141). With his extensive experience of nematology in SSA, Bridge (19) therefore considered that nematodes were rarely recognized as major limiting factors until all other constraints on yield increase had been removed. Nematode infection undermines resistance to other pests and diseases and also predisposes plants to other pathogens that, in turn, magnify losses but also mask the nematode damage. Various studies have demonstrated higher levels of fungal and bacterial root rot damage on root and tuber crops when coinfected with nematodes (37), although, in general, knowledge of nematode-disease-host interactions remains scarce in SSA.

Some key cities in SSA are among the fastest growing in the world, amplifying food demands, especially for fresh, perishable vegetable products. Peri-urban and urban vegetable systems emerge in response (see sidebar titled Vegetable Peri-Urban Systems). These small-scale but intensive systems are characterized by significant pest and disease pressure (2) and unsustainable production practices (102, 104, 118). The limited ability to identify the causal agent(s) of crop problems and the consequent misdiagnosis (126) lead to unskilled and indiscriminate use of pesticides, which compromises consumer food safety, farmer health, water supplies, and the environment (47, 90, 95, 152). Nowhere in SSA is the trade-off between the need to intensify agricultural production and protection of the environment as critical as in these systems. As nematode-infected roots deteriorate, host health is affected, resulting in greater pest and disease pressure (64) and increased reliance on pesticides (71). A complex of nematode species occurring simultaneously (11) further complicates the management of these pests. The use of genetic resistance is limited, noneffective pesticide use is high, and transmission through contaminated seedlings is rife (142). In order to sustainably produce and intensify vegetable production for these emerging mega-cities, major changes to production practices are required. Although there are more commercial farms supplying produce to cities, their sustainability and the intensification of smallholder peri-urban systems are dependent on accurate pest and disease information and knowing how to use this information to implement good agricultural practices (2, 152). Simple but effective mechanisms, such as the use of healthy seedlings and host resistance, can reduce pest and disease problems and consequently farmer’s reliance on pesticides (129). Peri-urban farmers in SSA almost exclusively establish their seedling nurseries in infested fields, generating RKN- and other soilborne pathogen–infected seedlings.

Vegetable Peri-Urban Systems

Root-knot nematodes (RKNs) are a malignant soilborne curse to vegetables, persistently undermining crop production. They are a particular concern because few farmers are aware of them or the damage they cause. In tropical regions, they are considered among the most important biotic constraints to vegetables (46). Other nematode species also occur, such as *Pratylenchus* spp. (lesion nematodes) and *Rotylenchulus reniformis* (reniform nematode), but *Meloidogyne* spp. are the most important. As nematode infection undermines resistance to other pests and diseases, it can directly lead to additional and inappropriate use of pesticides (71). Growers must tackle a pest and disease assault, and any export-bound produce needs to meet stringent pesticide residue standards. Synthetic pesticides are commonly used by producers to protect vegetables; however, correct identification of the causal reasons for crop damage are essential for proper use.

**Figure 1:**
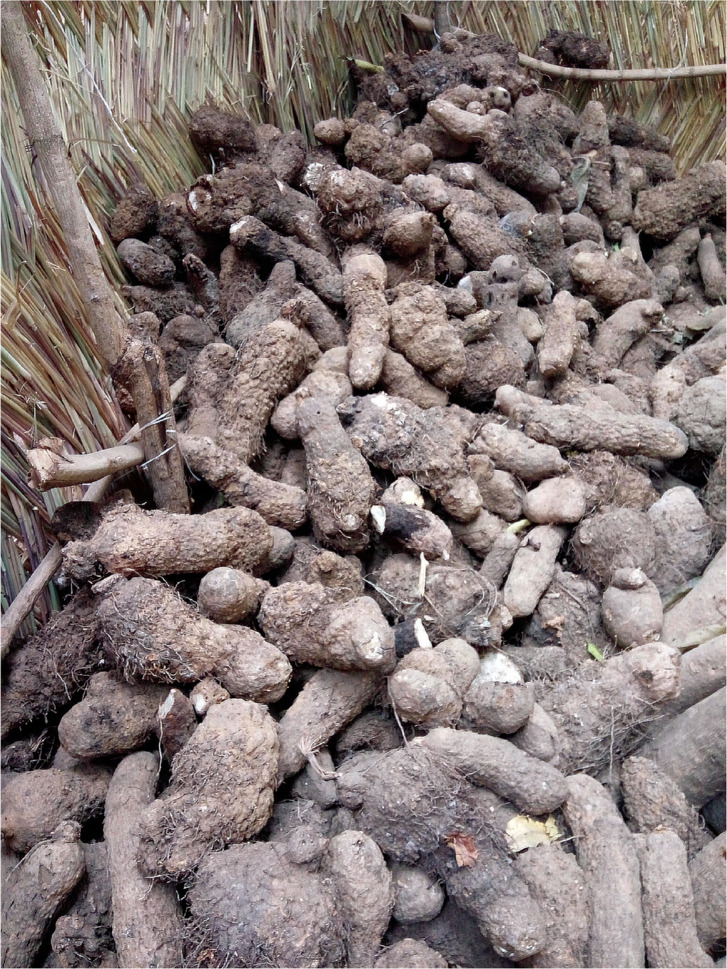
Recently harvested yams disfigured and galled due to root-knot nematode infection. Photo: Beatrice Aighewi.

In West Africa, yam (*Dioscorea* spp.) is an important staple food crop. Of two nematode groups that affect yam [see sidebar titled Yam (*Dioscorea* spp.)], *Scutellonema bradys* has traditionally been the more important, as opposed to RKNs, which deform the tubers with galls (18, 74). There is growing evidence, however, of increasing incidence of RKNs on yam and higher levels of damage (87), with some alarming wholesale rejection due to deformed tubers (**Figure 1**). The yam nematode is largely tuber-borne, whereas RKNs can be either tuber or field borne. Serial cultivation of yam on the same fields, with shorter intervals between crops, facilitates the escalation of RKN damage. However, a concomitant shift in the *Meloidogyne* species infecting the crop may also be responsible for increased levels of damage. For example, the aggressive RKN species *Meloidogyne enterolobii* was recently recovered from yam for the first time in Nigeria (88). Whether this species or a combination of other RKN species with *M. enterolobii* is leading to higher levels of galled tuber damage remains to be determined.

Although repeated cropping of the same land exacerbates nematode infestation (21), documented evidence of this is limited for SSA. Nonetheless, nematodes were identified as the most important threat to intensifying maize production systems in the Guinea Savannah of West Africa (147), despite a lack of focus on nematode issues (see sidebar titled Maize). On upland rice in Côte d’Ivoire, however, the number of sequential crop cycles on the same fields (*N* = 136) was directly correlated with *Pratylenchus* spp. and total nematode densities (41). Although this is not surprising (145), the rapid, exponential rise in cyst nematode (*Heterodera sacchari*) population densities on upland rice in West Africa over just three seasons of consecutive rice cultivation was surprising, with an unexpectedly rapid rate of increase observed (40). *H. sacchari* also proved highly aggressive, causing serious losses (7, 39), and consequently represents a major constraint on the regional intensification of rice production. For perennial crops, rotation is impractical, and, for example, because nematodes are among the greatest threats to pineapple production, synthetic chemical nematicides are routinely used on commercial operations across SSA (44, 114). In Zululand, South Africa, preplant nematode control is essential, especially on sandy soils (114), whereas in Kenya preplant fumigation with 1,3-dichloropropene-Telone II is routinely used, despite its highly restricted status (110). *Pratylenchus brachyurus* and *Meloidogyne javanica* are the key target species, occurring mostly in combination but sometimes with *Rotylenchulus reniformis*. In South Africa, average yield increases of 34% were recorded after nematicide application (83), with increases of up to 367% observed (98) in infested soils, demonstrating the need for alternative management options on smaller-scale establishments or once Telone is withdrawn.

Yam (*Dioscorea* Spp.)

There are essentially two key types of nematode affecting yam: lesion nematodes and root-knot nematodes (RKNs). *Scutellonema bradys* (yam nematode) is the primary lesion nematode and the cause of dry rot disease of tubers. *Pratylenchus sudanensis* causes similar dry rot symptoms to yam in East Africa, as does *Pratylenchus coffeae* elsewhere in the world; strangely, *P. coffeae*, although present, does not affect yam in Africa. *S. bradys* is considered the most important species. Low levels of infection on seed tubers, which may go undetected, provide inoculum to the next crop, and lead to heavy losses of infected tubers during storage. RKNs have traditionally been less of a problem. *Meloidogyne arenaria*, *Meloidogyne enterolobii*, *Meloidogyne incognita*,and *Meloidogyne javanica* are the species associated with yield loss in yam. Although RKNs are also seedborne, significant infection occurs in the field. The intensive cultivation of yam (and other crops) on the same land leads to the buildup of *Meloidogyne* spp. Nematodes can be responsible for heavy losses through reduced quality of tubers.

Maize

Maize is the most important food crop in sub-Saharan Africa (SSA). Lesion nematodes and root-knot nematodes (RKNs) commonly occur on maize, causing significant damage to production. Numerous other nematode species may also be simultaneously present. Both RKNs and lesion nematodes will undoubtedly be present at the same time. Lesion nematodes are commonly associated with maize production and correlate with poor growth and yield. Infected roots present necrotic lesions, which reduce root mass and may eventually destroy the roots. *Pratylenchus brachyurus* and *Pratylenchus zeae* are the most common species, and *Pratylenchus delattrei*, *Pratylenchus hexincisus*, *Pratylenchus penetrans*, *Pratylenchus sefaensis*,and *Pratylenchus scribneri* are also reported. Cereals, including maize, are often mistakenly viewed as poor hosts of RKNs, likely because of the lack of the typical galling symptoms that *Meloidogyne* spp. cause. Single or combined populations of *M. incognita* or *M. javanica* can seriously affect yields with losses of ≥50% observed (105, 117). Nematode damage to maize can be considerable, especially during crop development, and is exacerbated under marginal and water-stressed conditions.

## Nematode Diversity and Crop Damage

Intensive use of the same area of land by the same crop or crops with similar genetic background exerts significant pressure for pest and disease selection and virulence. However, in SSA, nematodes can pose severe threats even on newly opened, previously uncultivated land. The complete failure of tomato due to RKNs was observed on freshly cleared land in Ghana (50), and heavy RKN damage was experienced on vegetables in Kenya where land had previously not been cultivated (70). These early reports reflect a succession of similar, often undocumented observations across the continent by the authors of this review and numerous others. The often-unregulated nature of seed supply systems and an inherent lack of awareness for planting material hygiene by farmers also facilitates the dissemination of nematodes to new fields, especially with vegetatively propagated crops. In SSA, banana, plantain, yam, and potato are vegetatively propagated staple food crops, commonly cultivated by smallholders. The use and informal exchange of infected planting material perpetuate nematode infestation (133). Where tissue culture plants are not yet used, it is presumed that every single banana or plantain (*Musa* spp.) plant is infected with nematode pests to varying degrees (35).

Banana and Plantain (*Musa* Spp.)

*Radopholus similis* (burrowing nematode) is traditionally the most important nematode with respect to *Musa* cultivation because of its distribution, incidence, and aggressive nature (111, 113). However, except for on highland bananas in Eastern Africa, *R. similis* has gradually become less prevalent (38). *Pratylenchus goodeyi* (lesion nematode) is heat sensitive and customarily occurs at higher, cooler altitudes, but in Eastern and Southern Africa it is becoming more evident in tropically hot lowland areas. *Pratylenchus coffeae* has eclipsed *R. similis* in importance in West Africa and is becoming increasingly evident in Eastern and Southern Africa. *Helicotylenchus multicinctus* (spiral nematode) is commonly associated with *Musa* and in combination with RKNs is damaging to plantain, particularly in West and Central Africa. *Hoplolaimus pararobustus* (lance nematode) is a damaging species that is particularly large but occurs sporadically and in low densities. *Rotylenchulus reniformis* (reniform nematode) is being recovered increasingly from *Musa*, but its economic impact on *Musa* cultivation is as yet unknown. These species rarely occur in isolation, with complex infections of several species occurring regularly.

Banana and plantain are staple food crops with a clearly designated nematode problem [see sidebar titled Banana and Plantain (*Musa* spp.)], which can be used to demonstrate the impact of nematodes on crop production in SSA. Feeding by nematodes creates root necrosis and death, damaging the root system and weakening plant anchorage. Infected root systems systematically undermine bunch weight but when roots become severely damaged, their ability to support bunch-bearing stems is jeopardized, and during storms plants topple over with a total loss of bunches. In Côte d’Ivoire, Vilardebó (143) reported that in the first fruit cycle, the application of nematicides increased yields up to 211% and that *Radopholus similis* was causing average yield reductions of 80% in dessert banana plantations. Fogain (55) also recorded high overall yield losses of 60% on plantain production in Cameroon, and smallholder farmers have increasingly come to view plantain crops as single-cycle, as opposed to perennial, crops because of nematode infection (36, 103, 119). This reduced crop longevity, or number of cycles, has major implications, impacting on farmer returns and, importantly, on farmer decisions on whether or not to cultivate plantain. Yam (*Dioscorea* spp.), another vegetatively propagated staple crop is also persistently dogged by nematode problems [see sidebar titled Yam (*Dioscorea* spp.). As with *Musa*, the awareness and use of healthy planting material by farmers would resolve many of these issues (29), but because of various factors, including infrastructure, policy, and logistical and financial considerations, it is difficult to make significant changes over short time frames (6). Broad-scale implementation of sustainable healthy seed supply systems is necessary to effectively address this issue. Such a saturation of the system needs to be financially possible and ultimately acceptable by farmers, but saturation is a lengthy process fraught with obstacles. Awareness is necessary for farmers to understand the underlying causes of the problem and why it is important and financially beneficial to use healthy planting material.

Cassava, probably best known for its ability to withstand most afflictions, is generally viewed as being unaffected by nematodes (48). As a semiperennial that is often harvested piecemeal by smallholders, nematode damage is rarely observed (31). The naturally knobbly roots conceal RKN galling damage, and roots infected early in the season appear to deteriorate and die; thus, at harvest there are few infected roots remaining, with few nematodes present to correlate with crop growth, even though yield losses may be substantial (34). Coyne (32) estimated that 17% of Uganda cassava producers (*N* = 88) were subject to 66% yield losses owing to RKNs, whereas farmers in Nigeria were experiencing between 25% and *>*200% increase in cassava yields following solarization of fields infested with RKNs and *P. brachyurus* (5).

High-value crops, where demand for quality and quantity is market driven, can often influence practices and standards and in some cases can illustrate the importance of nematodes, especially where cosmetic appearance can be crucial. Potato provides one such example; it is important across SSA, where supply to the rapidly expanding fast food and snack industry is affected by RKNs, as a staple food crop. Tolerance levels for damage are strict. In South Africa, RKN damage is the main reason for downgrading potatoes at market, with more than a quarter of all downgrades due to RKNs (130). The processing industry accounts for approximately 20% (*>*$US80 million), and even though visually affected tubers are removed at the farm, a further 9% (˜$US8 million worth) of tubers presented to market are still downgraded (76). In Brazil, coffee plants are seriously affected by RKNs, with fields having been completely abandoned because of RKN damage (92, 99). In certain areas, coffee cultivation is only possible following grafting onto rootstock, which is resistant to RKNs (13), and some states actively enforce by law seedling certification for the absence of *Meloidogyne* spp. (144). However, in SSA, where coffee constitutes a major cash crop commodity, the impact of nematodes has, curiously, yet to be appreciated. There is scarce information on nematode species distribution and virtually none on their impact on production. Compared with coffee producing areas elsewhere (15, 24), it can be assumed that nematodes are causing significant damage to coffee production, which, consequently, would benefit from more attention to nematode management (e.g., 20).

## Root-Knot Nematodes (*Meloidogyne* Spp.)

Considering the entire range of crops affected, their distribution and prevalence, no nematode pest can approach the immense economic importance of *Meloidogyne* spp. (22, 109, 124, 132, 136, 137). This pervasive group of nematodes is widely distributed across SSA and the tropics, infecting most, if not all cultivated crops (109, 125). They are particularly destructive and, consequently, the authors view RKNs as probably the greatest biotic threat to agriculture in SSA, if not the entire tropics. Several species belong to a closely related, asexually reproducing (apomictic, mitotic parthenogenetic) complex known as the *M. incognita* group (MIG). These tropical RKNs include *Meloidogyne arenaria*, *M. incognita*,and *M. javanica*, which are believed to share a cryptic hybrid origin (26, 94). Although an asexual lifestyle often correlates with reduced adaptability to environmental variation and competition, MIG species are highly polyphagous and demonstrate substantial variation in virulence and aggression (131, 137, 153). The surprising success of MIG species may relate to a curious hybrid genomic structure that facilitates adaptive phenotypic plasticity (16). Alongside this, they are difficult to identify and have high fecundity and short generation times, all of which underpin their rapid spread (127). When examining the global spread of crop pests and pathogens, *M. incognita*, *M. javanica*,and *M. arenaria* are all notably among the most rapidly spreading pests (14).

The identification of RKNs from locations where resources for morphological or molecular analysis are limited or inaccessible has regularly resulted in the identification of *Meloidogyne* (and other genera) to genus only, or they are conveniently recorded as *M. incognita* (or *M. javanica*) (108). Our knowledge of the true identity and diversity of RKNs in SSA has, therefore, likely been obscured. Nematode diagnostic capabilities in SSA remain limited in general, although the situation is gradually changing as expertise develops and more reliable molecular techniques become available. For instance, using molecular mitochondrial haplotype techniques (72), *Meloidogyne oteifae* and *Meloidogyne decalineata*, previously recovered and described from coffee (51, 150), have now been synonymized with the redescribed *Meloidogyne africana*. Although it is intriguing that more nematode diagnostic work has not been undertaken in the context of coffee cultivation (51, 149–151), it is interesting that two African species, *M. africana* and an undescribed species from Tanzania (72), together with *Meloidogyne coffeicola* (in the Americas), are early branching species within this genus, suggesting that coffee may have played a key role in the evolution of RKNs. This basal position is of crucial importance in determining the ancestral characteristics of RKN reproduction, virulence, and morphology (26). Perhaps further investigations on RKNs and coffee cultivation will elucidate an improved understanding of this important group of pests.

Efforts to develop more reliable diagnostics for tropical MIG species were supported with RKN samples from across SSA to help determine species diversity, prevalence, and distribution (73, 108). These and other recent studies confirm that *M. incognita* and *M. javanica* are overwhelmingly the most prevalent species (73, 79, 86, 106, 108, 136). *M. enterolobii* occurs predominantly in West Africa but is also found sporadically across East and Southern Africa. Why there is such a disparity in distribution is not clear. However, although its increasing presence may be due to our improved ability to detect it, the increasing detection of this species may also partly be due to its pathogenicity on hosts resistant to other MIG species (25). As MIG species often occur in combinations, host resistance against one species can be of little use if other species against which the resistance is ineffective are present. Such is the case with the *Mi-1.2* resistance gene introgressed from *Solanum peruvianum* against RKNs in tomato (*Solanum lycopersicum*), which is effective against *M. aranaria*, *M. incognita*,and *M. javanica* (30) but not *M. enterolobii* (84). Consequently, full resistance against all the *Meloidogyne* species is needed for effective control where multiple species regularly occur simultaneously.

**Figure 2:**
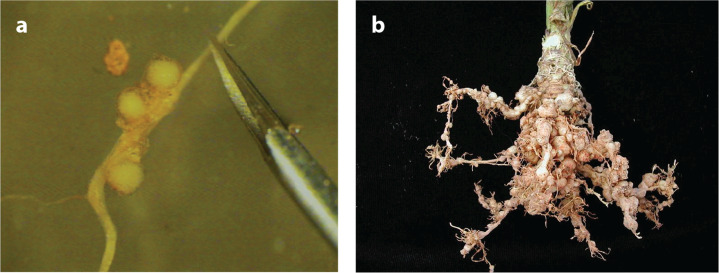
(*a*) Maize root infected with *Meloidogyne incognita*, showing the swollen female and egg sac protruding from the root but without any obvious galling or deformation of the root, unlike the (*b*) infected knotted mass of celery root.

**Figure 3:**
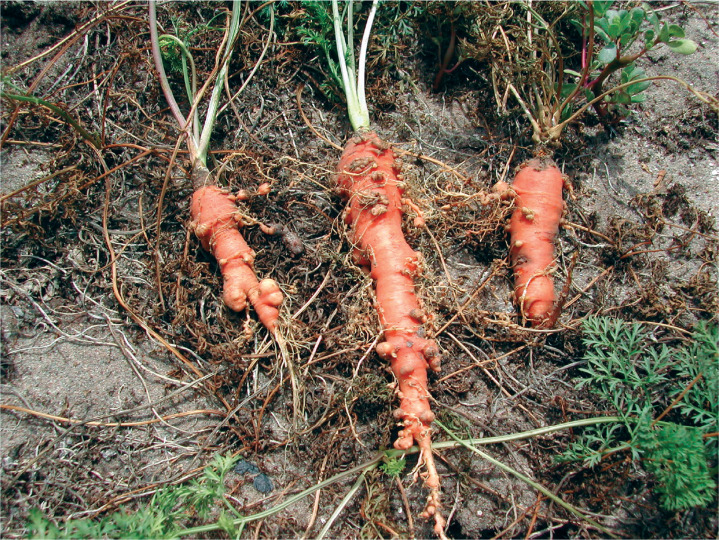
Carrots grown in sandy coastal soils under intensive peri-urban systems in Benin and deformed by *Meloidogyne* spp., resulting in the loss of the whole crop.

Relative to the scale of the problem, there are comparatively few documented examples of RKNs causing major losses from SSA for various reasons, including multiple nematode species infections combined with disease infections as well as limited awareness by farmers and observers. Vegetable crops, especially when grown under the intensive peri-urban systems, are systematically subjected to RKN challenge (see sidebar titled Vegetable Peri-Urban Systems), which is considered the single greatest biotic constraint to vegetable production, at least in West Africa (71). Severe crop damage has been consistently witnessed by the authors, where severely impaired root systems become unable to support the host and result in total losses (**Figures 2** and **3**). The negative impact of nematodes on yield affects commercial operations in addition to smallholders. Under highly managed conditions in Ethiopia, high-yielding hybrid tomatoes grafted onto imported nematode-resistant rootstock quickly became uneconomical and needed to be destroyed following RKN infection (D.L. Coyne, unpublished observations). The rootstock was evidently not resistant to the species present, demonstrating the importance of accurate, RKN species–level diagnosis. This is particularly crucial for those horticultural crops where natural plant resistance *R* genes control only certain species of RKNs, such as *Mi-1.2* in tomato and *Me1* and *Me3* in bell pepper (*Capsicum annuum*) (54). More stringent regulation of agricultural supply chains and support systems is required to equip farmers at all levels to make appropriate management decisions.

## Emerging Threats to African Agro-Ecosystem Resilience

As we broaden our investigations, introduce new crops or cultivars, and intensify cropping systems in the face of changing climates, new crop hosts and country records for nematode pests will arise. Improved diagnostics and nematology capacity will facilitate the discovery of these pests as well as identify new, undescribed species and aid in the redescription of existing species (46). For example, new reports of *M. enterolobii* from yam (88), African nightshade (27), sweet potato (79), and cowpea (86) have emerged from Nigeria, Kenya, and Mozambique, respectively, and undescribed species continue to surface (e.g., 73, 79, 108). Two other notably important RKN species in the United States and Europe, *Meloidogyne chitwoodi* and *Meloidogyne fallax*, have been formally identified in South Africa (57, 58). Anecdotal evidence indicates that *M. chitwoodi* may be present elsewhere in SSA (S. Haukeland, unpublished observations). Other recent discoveries include *Meloidogyne izalcoensis*, a highly damaging species on coffee, whose distribution was thought to be restricted to just a small isolated area of El Salvador (23) before its unexpected discovery in Tanzania, East Africa, on coffee and on cabbage and tomato in Benin, West Africa (77). Its impact on these crops in SSA is as yet unknown. Meanwhile, *Meloidogyne hapla*, a facultatively parthenogenetic (automictic) species with a temperate distribution, has been recovered from various temperate locations in Africa (106) but is now being recovered from more tropical conditions, such as in Benin (A. Affokpon, unpublished observations), Ethiopia (97), Kenya (G. Kariuki, unpublished observations), and Tanzania (72).

Species redescription will inevitably occur as diagnostic capabilities and infrastructure improve, as demonstrated by the mix-up of early RKN identifications on coffee, which was originally based on morphometrics alone (72)*. Pratylenchus speijeri*, a destructive lesion nematode on plantain (17), was also first studied and reported as *Pratylenchus coffeae* before its redescription based largely on molecular characteristics (45). Given that the diagnosis of *Pratylenchus* species on *Musa* has primarily relied on morphometrics alone, the curious and dramatic shift in *Pratylenchus* species dynamics on *Musa* in both East and West Africa [see sidebar titled Banana and Plantain (*Musa* spp.)] raises questions regarding their true identity. There are considerable difficulties associated with differentiating species of *Pratylenchus* morphometrically, especially with the *P. coffeae* sensu lato group (49). The nematode species occurrence has altered radically in some places from *R. similis* to *P. coffeae* (33), although elsewhere the ecological range of *Pratylenchus goodeyi* appears to have adjusted and is now present in hot tropical locations in Tanzania (93) and Kenya (123), where it was previously undetected (63, 112, 116). In West Africa, *P. coffeae* has replaced *R. similis*,which has traditionally been the dominant nematode pest; in East Africa, *P. coffeae* incidence is rising (93, 123). Uncertainties concerning the validity of nematode species, unless resolved, will present practical problems related to their management and quarantine measures (46), a point highlighted by the recent discovery of PCNs in SSA. Indigenous to South America and since introduced to various potato growing regions of the world, PCNs had effectively not been recorded in SSA until they were confirmed in Kenya in 2015 (96, 100) (see sidebar titled Potato). This will quite likely prove to be the gateway for PCNs regionally and across SSA, and advice and support will be necessary to lessen the devastating effects of PCNs on potato; an old adversary, now present and new to SSA. Porous national borders and limited expertise within national phytosanitary services will hinder efforts to contain the problem. Meanwhile, multiple potato crops per year on the same area, subtropical temperatures, and farmer reticence to uproot volunteer potato plants will lead to high and damaging population densities.

Potato

Numerous nematode species and genera are reported from potato. In sub-Saharan Africa (SSA), root-knot nematodes (RKNs) and lesion nematodes appear the most important, although information on the overall diversity and impact of nematodes on potato is limited. *Meloidogyne* species, such as *Meloidogyne hapla*, *Meloidogyne incognita*,and *Meloidogyne javanica*, are probably the most threatening, although they may occur in combination with *Pratylenchus* spp., as well as other species. *Ditylenchus* spp., *Paratrichodorus* spp., *Scutellonema bradys* and *Trichodorus* spp. are other potential threats. Globally, the most important nematodes affecting potato are potato cyst nematodes (PCNs) (*Globodera rostochiensis* and *Globodera pallida*) (138), which are major quarantine pests and now present in Kenya (100) in all major potato growing areas (69). The discovery of a new species of PCN in the United States, *Globodera ellingtonae* (66), illustrates the importance of a constant vigil for new nematode pests. A major challenge is that eggs, encased in cysts, are able to survive in the soil without a host for years. Together with RKNs and lesion nematodes, which both have multiple hosts, nematode management in potato will become a significant challenge under more intensive cultivation. The impact of RKNs can be high through reduced quality of tubers.

Internationally, policies are in place for the detection and reporting of quarantine and invasive pests under an international framework (**https://www.ippc.int/en/**). However, global trade and the increasing movement of contaminated plant material places constant pressure on national efforts to prevent the entry of new pests (14, 46). The example of PCNs in Kenya is a clear indication of how a pest that is subject to strict quarantine regulations worldwide (52) can readily become established in a country where nematology expertise has only recently been made available in the national phytosanitary service. The views that PCNs are temperate pests and that nematode pests of potato in the region were considered unimportant have not helped (60). This oversight has proved a hard lesson and a strong justification for strengthened nematology expertise within phytosanitary services.

## Climate Change

Climate change is predicted to have a severe impact on Africa (68). Depending on the model, temperatures are predicted to rise between 1.5°Cand5.0°C above the 1951–1980 baseline by 2100. There is much concern surrounding the impact climate change will have on pests, diseases, and crop productivity, and, consequently, how we need to adapt to cope with these changing threats. In respect to nematodes, there is limited evidence of what the impact will be. We can speculate on the basis of known climatological ranges of nematode species, but given our limited knowledge in general on species distribution, temperature thresholds, and survival under different climates, there will be much guesswork and many assumptions. Recent studies on *M. incognita*, *M. javanica*,and *M. arenaria* and different geographic populations of these species, demonstrated that these pathogens have remarkable behavioral plasticity (J. Dalzell, unpublished observations). Different populations demonstrated a high degree of variability in aggression and infectivity between temperatures. This creates difficulties in determining pest population reactions to such environmentally influential determinants, which will also relate to changes in crop root exudate profiles (10, 59, 85) that trigger hatching and attraction of some nematode species. Within the tropics, various nematode species have likely reached their life-cycle thermo-optima, but as the range of subtropical to temperate climates prevails within SSA, rising temperatures will influence nematode pest dynamics in these agro-ecologies, resulting in shortened life cycles and more rapid pest buildup. It is difficult to predict the outcomes, however, as myriad permutations occur under such dramatic environmental changes, with a multitude of possible implications. One example is available to illustrate potential effects: bananas in the Eastern African Highlands. Banana is affected by a number of nematode species [see sidebar titled Banana and Plantain (*Musa* spp.)]; *R. similis*,the most damaging species, is dominant up to approximately 1,400 m above sea level (35, 80). Above this altitude, *P. goodeyi*, a less-damaging species, replaces *R. similis*, creating a thermo-regulated segregation of the two species, based on their respective temperature preference ranges (128). The altitudinal range of *R. similis* will thus rise with a rise in temperature, subjecting more bananas to the more aggressive nematode; a 1°C rise in temperature corresponds with a 170-m altitude change (D. Ochola, unpublished observations).

In order to cope with a gradually warmer SSA, it becomes increasingly important to optimize water-use efficiency and to make better use of existing water (82). Although multidisciplinary approaches advocate developing more water-efficient crop cultivars for water-stressed conditions, it appears more consideration of biotic aspects that affect root performance may be needed (e.g., see 89). Maize, although the most important cereal food crop in SSA, receives little attention with respect to breeding for nematode resistance (see sidebar titled Maize) even though nematodes can have a major influence on root performance and yields. In a study assessing resistance to drought and nematodes, the irrigation option × genotype interaction was shown to significantly affect maize growth. The study strongly recommended that certain genotypes be included in maize breeding programs as donors for *Pratylenchus zeae*/*M. incognita*–resistant and drought-tolerant genes (78), yet the research remained focused on drought resistance without including nematode resistance. Similarly, *Coffeae canephora* ‘Conilon’ was identified as a drought-tolerant clone in Brazil (43) and, coincidentally, in a separate study (91) was shown to be highly resistant to RKNs. To increase crop water-use efficiency, much more can be gained from addressing nematology aspects when breeding for more water-efficient genotypes. Undoubtedly, a key component of this nematology oversight reflects the paucity of nematology expertise. Raising the nematology profile with greater access to expertise and greater visibility through research publications and open access outlets will gradually aid greater disciplinary integration.

## Nematology Training and Capacity Building

In order to advance awareness of nematology in the agricultural arena in SSA, a fundamental shift in how this is approached is required, from basic information for the farmer, through academia, to scientific publications. The specialized capacity building targeting both farmers and national extension services has been highlighted as a strategic factor to boost agricultural productivity by smallholders in SSA (46), and similar overarching structural challenges have been identified elsewhere (3). Within SSA, nematology largely remains a semirooted discipline with only limited instances in which it is presented as a stand-alone topic. Although limited, this shift in curricula is significant. This can arguably be attributed to long-standing efforts pitched at building a critical mass of postgraduate and research scientists. One notable initiative, the Nematology Initiative for Eastern and Southern Africa, focused on building capacity, training more than 60 people in seven countries (61). The roles of nematological societies both within and outside Africa, in particular the Nematological Society of Southern Africa, are of course a source of support and inspiration to current and future nematologists. More nationally based societies, such as the Nigerian Society of Nematologists, also provide huge local motivation. In East Africa, the nematology platform developed through *icipe* (International Centre of Insect Physiology and Ecology)/IITA (International Institute of Tropical Agriculture) has created a highly active network of individuals and activity in the region. Without a doubt, however, the specialized nematology training at Ghent University, Belgium is unrivaled in its provision of nematology expertise to SSA. Since 1992, 168 students from 20 African countries, mostly from western and eastern Africa, have graduated in nematology from Ghent University. To further understand the status of nematology in SSA, an online survey of 125 individuals (L. Cortada, unpublished observations) revealed that most nematology training is gained at the MS level and least at the BS level. Ghent University was the main source of nematology knowledge (55% respondents), followed by national universities (39%), IITA (23%), international universities (14%), and *icipe* (8%). Encouragingly, 56% of respondents were able to pursue a nematology-related profession, 68% returned home, and an impressive 30% gained a PhD.

Despite the low number of nematology publications (filtered search at Scopus using “plant parasitic nematodes”), it has risen steadily from one manuscript per year in 1972 to a recent maximum of 25, although they tend to be published in specialized journals (e.g., *Nematology* and *Nematropica*). In order to stimulate wider and greater multidisciplinary interaction, more critical mass in the general scientific literature and other open access journals is crucial.

Although there has been much improvement regarding nematology within university curricula and in available expertise in SSA, evidence indicates that this has not necessarily translated into increased awareness at the national government level (4, 132). Consequently, capacity building across the agricultural spectrum is required to improve the productivity of smallholders, ideally under a decentralized and unpoliticized extension system involving the private sector (154) and with adequate coordination, motivation, and financial support that also enable the agricultural policies already in place (115).

## The Future Outlook

Since the International *Meloidogyne* Project (121), there has been no other undertaking of a comparable scale that has addressed the enormity of the nematode problems in SSA and the tropics. Although nematologists may lament the lack of support apportioned to nematode issues, it ultimately rests on our shoulders to attract the relevant attention and support to address the seriousness of the issue. This requires a measured, progressive approach involving the elevation of awareness across the governmental, academic, and donor sectors as well as in farmers and the public at large. It also requires a proportionate demonstration of the damage caused by nematodes and their future impact. This takes time, and the complexity of smallholder farming systems, under a predominantly tropical climate, compounds the situation. However, in attempting to predict future scenarios, Sikora et al. (124) recognized a bright and successful future for nematology in the tropics and subtropics.

Within this review, we have tried to project an image of the magnitude of nematode infestation in SSA but also of the peculiarities of the region that create difficulties for their management. For progress to be made, we have emphasized training and awareness in nematology as well as the shift that is taking place in recognition of this. We did not dwell on management options, but for better progress, it is clear that suitable, adoptable, and relevant options need to be made available. Simple techniques, such as the disinfection of *Musa* planting material by dipping it in boiling water, have been developed, are available, and are being adopted by farmers (42, 67, 133). Integrating their use across SSA, however, requires significant logistics and finances. Healthy planting material is critical to reducing the impact of nematode pests, as is the use of host resistance, and biological control is attracting increasing attention. For this to have any impact, however, quality diagnostics and access to suitable resistant cultivars and quality biological control products are required. Despite the fact that current policies tend to discourage investors from registering new products, there is surprising interest from both national and international companies to bring new biologically based products onto the market. Multiple nematode infections can also render host resistance useless if resistance against the whole range of nematode species present is not conferred. The widespread distribution of aggressive and highly virulent, resistance-breaking nematode strains across SSA also demands that several resistance traits are available within each new crop cultivar.

In the not too distant past, Gressel et al. (65) assessed biotic constraints in Africa that could be addressed through biotechnology. No mention was made of nematodes as a biotic constraint to crop production or as a target for biotechnological interventions. Likewise, Thomson (134) does not include nematodes as a major concern or target of biotechnological intervention. There are no shortages of realized and potential approaches to interfere with nematode parasitism using modern crop improvement tools for cost-effective and reliable options (8). A substantial body of literature has developed around the use of RNA interference to knockdown the expression of nematode genes in planta (12). Several crop RNAi traits have now been commercialized in Canada and the United States. The development of antifeedant strategies that employ cystatins has proven successful in isolation and in combination with more recent peptide traits that interfere with nematode sensory perception (135). Rhizosphere microbes have been engineered to synthesize and secrete peptides that interfere with nematode host-finding (146). Such approaches to engineering the rhizosphere for nematode control avoid the need to modify the food crop directly, which may lower sociopolitical barriers to adoption. Developing crop host resistance through biotechnology also enables the use of cultivars that are already preferred by farmers (135). It also enables the stacking of resistance traits for use against multiple nematode species. Use of farmer-preferred cultivars is especially important for traditional crops, such as *Musa*, that are otherwise diffi-cult to breed and can take a considerable amount of time to develop new cultivars (107). For example, conventional breeding in these traditional crops takes, on average, 20 years (**http:// breedingbetterbananas.org/**). If nematode profiles that are affected by different sources of resistance are similarly changing within this lengthy time frame, then this seriously compromises such efforts. With shifting nematology profiles playing out across SSA, as demonstrated by *Musa*, resistant breeding programs need to be as robust as possible and account for as broad a range of nematode species as feasible. The ability to target several species through transgenic approaches would enable more efficient and timely deployment of novel sources of resistance (9).

A substantial component of future-proofed nematology must be a more holistic integration of nematodes into a broader and more inclusive narrative around crop improvement strategies, training programs, and policies for SSA. Success in addressing nematode problems in SSA will lie in embracing novel biotechnology juxtaposed with the use of simple, effective mechanisms. Undoubtedly, our ability to create public awareness and understanding across the agricultural landscape will ultimately determine progress. In our experience, the establishment of networks or platforms, such as in Kenya and Nigeria, provide a critical mass of nematology expertise, which generates activity and publicity while providing support to the region. The outlook is positive and optimistic, albeit with progress slower than we would prefer.

Summary Points

1. Nematology remains a neglected and semirooted discipline across SSA, largely because of limited technical capacity, awareness of local stakeholders (public and private), and availability of specific resources dedicated to it; however, with the increasing inclusion of nematology within the curricula of African national universities, this is gradually changing.
2. Nematode infection is more often a combination of multiple species and strains, creating difficulties in assessing the importance of individual species and hampering the use of effective nematode management options.
3. African smallholder cropping systems are complex and often characterized by a simultaneous and continuous cultivation of a diversity of crops, providing conditions for multiple generations and rapid population buildup.
4. RKNs (*Meloidogyne* spp.) represent the major nematode pest threat and probably constitute the greatest biotic threat to productivity across the continent and tropics.
5. There is an immense need to intensify crop production systems to increase production, which will exert increasing pressure on the selection of nematode pests and virulence.
6. New and emerging threats, such as PCNs in East Africa, paradoxically provide greater visibility and publicity to nematode issues.
7. With climate change, we will likely see greater nematode damage across SSA.
8. Current conditions for registration of new biologically based products that are environmentally safer than existing chemical pesticides are discouraging for investors.

Future Issues

1. Although nematology expertise is becoming increasingly available, awareness and knowledge have yet to adequately reach national governments and this needs to be addressed. Policy changes also must occur to provide better private and public opportunities for managing nematodes.
2. Farmers need to gain greater awareness of nematode problems to address the issue.
3. To stimulate wider and greater multidisciplinary interaction, more critical mass in the general scientific literature and other open access journals will be necessary.
4. Increasing access to and reducing costs of molecular diagnostic techniques will enable better and more rapid characterization of nematode pests to enable more balanced decisions for pest management.
5. Novel biotechnology tools are a means to providing cost-effective and reliable nematode management options.
6. RKNs (*Meloidogyne* spp.) will become a more pervasive threat to crop production without the required attention they deserve.
7. The involvement of nematodes in pest and disease complexes requires much better attention to help elucidate the underlying causes of crop losses and how to deal with them.
